# The Performance of ChatGPT on the American Society for Surgery of the Hand Self-Assessment Examination

**DOI:** 10.7759/cureus.58950

**Published:** 2024-04-24

**Authors:** Sebastian D Arango, Jason C Flynn, Jacob Zeitlin, Daniel J Lorenzana, Andrew J Miller, Matthew S Wilson, Adam B Strohl, Lawrence E Weiss, Tristan B Weir

**Affiliations:** 1 Department of Orthopaedic Surgery, Philadelphia Hand to Shoulder Center, Philadelphia, USA; 2 Department of Orthopaedic Surgery, Sidney Kimmel Medical College, Philadelphia, USA; 3 Division of Orthopaedic Hand Surgery, OAA Orthopaedic Specialists, Allentown, USA

**Keywords:** self-assessment examination, hand, chatgpt, assh, artificial intelligence

## Abstract

Background: This study aims to compare the performance of ChatGPT-3.5 (GPT-3.5) and ChatGPT-4 (GPT-4) on the American Society for Surgery of the Hand (ASSH) Self-Assessment Examination (SAE) to determine their potential as educational tools.

Methods: This study assessed the proportion of correct answers to text-based questions on the 2021 and 2022 ASSH SAE between untrained ChatGPT versions. Secondary analyses assessed the performance of ChatGPT based on question difficulty and question category. The outcomes of ChatGPT were compared with the performance of actual examinees on the ASSH SAE.

Results: A total of 238 questions were included in the analysis. Compared with GPT-3.5, GPT-4 provided significantly more correct answers overall (58.0% versus 68.9%, respectively*;* *P *= 0.013), on the 2022 SAE (55.9% versus 72.9%; *P *= 0.007), and more difficult questions (48.8% versus 63.6%; *P* = 0.02). In a multivariable logistic regression analysis, correct answers were predicted by GPT-4 (odds ratio [OR], 1.66; *P* = 0.011), increased question difficulty (OR, 0.59; *P* = 0.009), Bone and Joint questions (OR, 0.18; *P* < 0.001), and Soft Tissue questions (OR, 0.30; *P* = 0.013). Actual examinees scored a mean of 21.6% above GPT-3.5 and 10.7% above GPT-4. The mean percentage of correct answers by actual examinees was significantly higher for correct (versus incorrect) ChatGPT answers.

Conclusions: GPT-4 demonstrated improved performance over GPT-3.5 on the ASSH SAE, especially on more difficult questions. Actual examinees scored higher than both versions of ChatGPT, but the margin was cut in half by GPT-4.

## Introduction

There has been an increase in the use of artificial intelligence (AI) in medicine over the past several years [1-7]. Large language models (LLMs), such as Chat Generative Pre-trained Transformer (ChatGPT; OpenAI, San Francisco, CA) [8], encompass a type of machine learning that analyzes text and synthesizes natural, human-like responses to promote dialogue between the user and the model [9]. ChatGPT-4 (GPT-4) is the successor of ChatGPT-3.5 (GPT-3.5) and represents the newest and most advanced version of ChatGPT to date [10]. By serving as a dialogic agent that mimics human language, ChatGPT creates an opportunity for a type of *small-group* learning that can be utilized by trainees, surgeons, and patients. With over 1,800 PubMed-indexed publications related to ChatGPT in 2023 and over 100 million weekly users, the technology is becoming increasingly utilized [8].

To serve as a viable learning tool and resource in hand surgery, the information generated by ChatGPT must be accurate, consistent, and reliable. Prior studies evaluating ChatGPT in hand surgery have focused on the qualitative responses to common patient questions [11,12]. In contrast, other studies have evaluated ChatGPT’s performance on examinations intended for trainees [3,9,10,13-16]. The American Society for Surgery of the Hand (ASSH) Self-Assessment Examination (SAE) is primarily focused on testing the knowledge of hand surgeons [17]. Since the ASSH SAE may be considered reflective of evidence-based hand surgery topics, the evaluation of the performance of ChatGPT on this examination may enable insights into AI’s current command of hand surgery knowledge. Although there have been two inquiries evaluating ChatGPT's capabilities on the ASSH SAE, which demonstrated that its proficiency does not yet meet the standards expected for hand surgeons, these examinations have solely focused on a singular version of ChatGPT [18,19]. Furthermore, they lacked a comparative evaluation of ChatGPT’s performance against human examinees answering the most recent SAE questions reflecting current advancements in evidence-based hand surgery [18,19].

Therefore, the primary purpose of this study was to evaluate the overall performance of GPT-3.5 and GPT-4 on the 2021 and 2022 ASSH SAE. The secondary aims were to compare ChatGPT’s performance based on question difficulty and question category. Finally, the performance of actual examinees was compared to both versions of ChatGPT. We hypothesized that GPT-4 would outperform GPT-3.5 by providing significantly more correct answers overall, question difficulty level, and question category. We anticipated that actual examinees would have a higher performance than either version of ChatGPT.

## Materials and methods

The ASSH SAE Committee is tasked with the creation of the annual ASSH SAE. The examination consists of 200 multiple-choice questions, with five answer choices per question. The SAE is offered in two examination formats that allow surgeons to start and stop the exam at their own pace. Examinees can choose to answer all questions before receiving the correct response and explanation to receive Continuing Medical Education (CME) and Maintenance of Certification (MOC) credit [17,20,21]. Alternatively, examinees can choose to receive the correct response and explanation as each question is answered to receive CME credit alone. Examinees are allowed to use outside resources to answer each question. Questions are written and peer-reviewed by board-certified hand surgeon members of the ASSH SAE Committee. The committee meets face-to-face twice annually to review and edit the questions created by each member over the year. The questions are intended to test the knowledge of hand surgeons and affiliates with timely information that is representative of the changing field of hand surgery [17]. The minimum passing score for CME credit is 50% for orthopedic and plastic surgeons and 75% for general surgeons. The SAE is commonly used to prepare for the Subspecialty Certificate in Surgery of the Hand, receive MOC/CME credit, and remain up to date with current topics in hand surgery.

Before initiating data collection, we obtained permission from the ASSH to perform the study. The 2021 and 2022 ASSH SAE questions were screened by a single reviewer to include text-only questions. Given the July 2023 version of ChatGPT was not capable of analyzing images or videos, questions with tables, images, and figures were excluded from the study. Each question was then formatted as a standalone multiple-choice question, retaining the original phrasing of the source material. ChatGPT was asked questions without prior training to determine a raw baseline performance level. All questions were asked in a new chat box to limit ChatGPT’s potential for providing answers based on information from prior question stems. Data collection was conducted over four days (July 18-21, 2023) to limit the influence of ChatGPT updates on the results. If ChatGPT provided more than one answer to a question, the phrase *choose the single best answer* was added to the question stem before being asked again in a new chat window. Questions for which ChatGPT could not provide a single best answer on the second attempt were considered incorrect. 

The primary aim of this study was to compare the proportion of correct answers between GPT-3.5 and GPT-4 on the ASSH SAE. The secondary aims were to determine if question difficulty or question category influenced the performance of ChatGPT. Question difficulty was graded by two raters according to cognitive taxonomic levels, as described by Buckwalter et al. [22]. Briefly, Level 1 questions are those dealing with the recognition and recall of discrete facts, Level 2 questions involve the evaluation of data comprehension and interpretation, and Level 3 questions are the most difficult and require the application of knowledge to resolve specific problems [22]. Questions were categorized according to the seven ASSH SAE question categories: Basic Science, Bone and Joint, Neuromuscular, Skin, Vascular, Ancillary, and Miscellaneous [17]. Finally, the ASSH SAE Committee provided the study with question-level statistics to determine the percentage of actual examinees that answered each question correctly. We then compared the mean percentage of correct answers by actual examinees to the performance of ChatGPT.

Statistical analysis

A sample size calculation was performed to detect a significant difference between the proportion of questions answered correctly by GPT-3.5 and GPT-4 based on a prior study [15]. Assuming GPT-3.5 answers 54% of questions correctly and GPT-4 answers 74% of questions correctly [15], an alpha error of 0.05, 80% power, and an allocation ratio of 1:1, a sample size of 180 questions was required. Given each ASSH SAE has 200 questions, and roughly 50% of the questions are text-based without figures or tables, it was determined that the two most recent examinations (years 2021 and 2022) would be sufficient to power the study. It was estimated that the two examinations would provide approximately 200 questions for analysis.

The counts with percentages were calculated and proportions were compared with chi-square or Fisher’s exact tests. Inter-rater reliability for question difficulty (taxonomy level) was determined by calculating the intraclass correlation coefficient (ICC) using a two-way mixed effects model. ICC values over 0.90 represent excellent reliability [23]. If the raters disagreed on a question taxonomy rating, a third rater served as a tiebreaker. A multivariable logistic regression analysis was performed to determine if GPT-3.5 or GPT-4, examination year, question difficulty (taxonomy level), or question category were predictive of a correct answer. Variables with a *P*-value < 0.20 on the univariate analysis were not included in the final multivariable model. Finally, the mean percentage of correct answers by actual examinees was compared with independent samples *t*-tests to determine if examinees performed better on questions answered correctly or incorrectly by ChatGPT. Differences with *P* ≤ 0.05 were considered statistically significant.

## Results

Of the 400 questions available for review, 238 questions (SAE 2021, *n* = 120; SAE 2022, *n* = 118) were text-only without associated figures or tables and were included in the analysis. Of the included questions, 117 (49%) were Level 1 (recall) taxonomy, 55 (23%) were Level 2 (interpretation) taxonomy, and 66 (28%) were Level 3 (application) taxonomy. The ICC for question taxonomy rating was 0.957 (95% confidence interval [CI], 0.944-0.966), indicating excellent reliability. The top 3 question categories were *Bone and Joint *(29%), *Neuromuscular* (19%), and *Miscellaneous* (19%). There were no significant differences in question taxonomy or category between the SAE 2021 and SAE 2022 examination years. Most actual examinees were ASSH Active Members (2,242, 58%), followed by Nonmembers (825, 21%) and Lifetime Members (341, 9%), accounting for 88% of the total examinees (Table 1). 

**Table 1:** ASSH SAE examination characteristics. Values are given as the number or as the number with the percentage in parentheses. ^†^Comparison between 2021 and 2022 ASSH SAEs. ^*^Statistically significant values with *P* ≤ 0.05. ASSH, American Society for Surgery of the Hand; SAE, Self-Assessment Examination

Variable	Total	Examination year		*P*-value^†^
		2021	2022	
Total questions	400	200	200	―
Questions without images	238	120	118	―
Question taxonomy				
Level 1 (recall)	117 (49)	59 (49)	58 (49)	0.82
Level 2 (interpretation)	55 (23)	26 (22)	29 (25)	
Level 3 (application)	66 (28)	35 (29)	31 (26)	
Question category				
Basic Science	22 (9)	10 (8)	12 (10)	0.91
Bone and Joint	69 (29)	39 (33)	30 (26)	
Neuromuscular	46 (19)	22 (18)	24 (20)	
Skin	22 (9)	11 (9)	11 (9)	
Vascular	18 (8)	10 (8)	8 (7)	
Ancillary	16 (7)	7 (6)	9 (8)	
Miscellaneous	45 (19)	21 (18)	24 (20)	
Examinee ASSH membership status				
Total	3,848	1,926	1,922	―
Active	2,242 (58)	1,133 (59)	1,109 (58)	
Allied Health/Affiliate	84 (2)	39 (2)	45 (2)	
Candidate	232 (6)	97 (5)	135 (7)	
International	44 (1)	21 (1)	23 (1)	
Lifetime	341 (9)	175 (9)	166 (9)	
Retired	71 (2)	40 (2)	31 (2)	
Resident/Fellow	9 (0)	4 (0)	5 (0)	
Nonmembers	825 (21)	417 (22)	408 (21)	

GPT-4 answered a significantly greater proportion of questions correctly compared with GPT-3.5 (GPT-4, *n* = 164, 68.9%; GPT-3.5, *n* = 138, 58.0%; *P* = 0.013). A second prompt was required for GPT-3.5 and GPT-4 to answer the question in 11 (4.6%) and 13 (5.5%; *P* = 0.68) questions, respectively. No answer was provided after two prompts for 2 (1%) questions in both GPT-3.5 and GPT-4. While there was no significant difference between ChatGPT versions on the SAE 2021 examination, GPT-4 answered significantly more questions correctly on the SAE 2022 examination than GPT-3.5 (72.9% versus 55.9%; *P* = 0.007). There was no significant difference in ChatGPT version performance based on direct recall (Level 1 taxonomy) questions, but GPT-4 correctly answered significantly more high-level taxonomy (Level 2 or Level 3) questions than GPT-3.5 (63.6% versus 48.8%, respectively; *P* = 0.02). There were no significant differences in performance based on the question category. The mean percentage of correct answers by actual examinees was higher than both ChatGPT versions overall, examination year, and question difficulty. Actual examinee performance ranged from 76.5% to 83.0% based on the question category, while GPT-3.5 (46.4%-81.3%) and GPT-4 (53.6%-95.5%) had greater variance (Table 2).

**Table 2:** ChatGPT-3.5 and ChatGPT-4 performance on the ASSH SAE. Values are given as the number, as the number with the percentage in parentheses, or as the mean percentage correct plus or minus the standard deviation. ^§^If ChatGPT did not provide a definitive answer on the first attempt, the question was asked a second time. ^†^ChatGPT did not provide a definitive answer on the first or second attempt. Unanswered questions were considered incorrect. ^‡^Statistical comparisons were calculated with Fisher’s exact or chi-square tests comparing GPT-3.5 and GPT-4. ^*^Statistically significant values with *P* ≤ 0.05. ASSH, American Society for Surgery of the Hand; SAE, Self-Assessment Examination; NS, not significant

Variable	Examinees	GPT-3.5, *n* (%)	GPT-4, *n* (%)	*P*-value^‡^
Correct answers	79.6 ± 18.3	138 (58.0)	164 (68.9)	0.013*
Second attempt required^§^	-	11 (4.6)	13 (5.5)	0.68
No answer on any attempt^†^	-	2 (1)	2 (1)	NS
Correct by examination year				
2021	78.1 ± 19.7	72 (60.0)	78 (65.0)	0.42
2022	81.1 ± 16.7	66 (55.9)	86 (72.9)	0.007*
Correct by question taxonomy				
Level 1 (recall)	80.3 ± 17.5	79 (67.5)	87 (74.4)	0.25
Level 2 (interpretation)	75.4 ± 20.2	26 (47.3)	35 (63.6)	0.084
Level 3 (application)	81.7 ± 17.8	33 (50.0)	42 (63.6)	0.11
Level 2 or 3	78.8 ± 19.1	59 (48.8)	77 (63.6)	0.02*
Correct by question category				
Basic Science	83.0 ± 12.8	17 (77.3)	21 (95.5)	0.19
Bone and Joint	80.4 ± 19.0	32 (46.4)	37 (53.6)	0.40
Neuromuscular	76.5 ± 17.7	27 (58.7)	29 (63.0)	0.67
Skin	79.7 ± 16.9	12 (54.5)	18 (81.8)	0.052
Vascular	78.3 ± 21.0	9 (50.0)	12 (66.7)	0.31
Ancillary	77.8 ± 21.4	13 (81.3)	14 (87.5)	NS
Miscellaneous	80.8 ± 19.3	28 (62.2)	33 (73.3)	0.26

A multivariable logistic regression analysis showed GPT-4 was 66% more likely to answer questions correctly than GPT-3.5 (odds ratio [OR], 1.66; 95% CI, 1.13-2.46; *P* = 0.011). Higher taxonomy questions were significantly associated with incorrect answers, where Level 2 or 3 taxonomy questions were 41% less likely to be answered correctly than Level 1 questions (OR, 0.59; 95% CI, 0.40-0.88; *P* = 0.009). Finally, the question category was significantly predictive of incorrect answers, where *Bone and Joint* questions were 82% less likely to be answered correctly compared with *Basic Science* questions (OR, 0.18; 95% CI, 0.07-0.46; *P* < 0.001) and *Soft Tissue* questions were 70% less likely to be answered correctly (OR, 0.30; 95% CI, 0.12-0.78; *P* = 0.013; Table 3).

**Table 3:** Multivariable logistic regression for predictors of correct answers. ^†^Soft Tissue questions include Neuromuscular, Skin, and Vascular categories. ^‡^Other questions include Ancillary and Miscellaneous categories. ^*^Statistically significant values with *P* ≤ 0.05. OR, odds ratio; GPT, Generative Pre-trained Transformer; CI, confidence interval

Variable	Univariate		Multivariable	
	OR (95% CI)	*P*-value	OR (95% CI)	*P*-value
GPT-4, Ref. = GPT-3.5	1.61 (1.10-2.34)	0.014*	1.66 (1.13-2.46)	0.011*
Exam year 2022, Ref. = 2021	1.09 (0.75-1.58)	0.67	-	-
Taxonomy				
Level 1	Ref.	-	Ref.	-
Level 2 or 3	0.53 (0.36-0.77)	<0.001*	0.59 (0.40-0.88)	0.009*
Question category				
Basic Science	Ref.	-	Ref.	-
Bone and Joint	0.16 (0.06-0.40)	<0.001*	0.18 (0.07-0.46)	<0.001*
Soft Tissue^†^	0.26 (0.10-0.65)	0.004*	0.30 (0.12-0.78)	0.013*
Other^‡^	0.41 (0.16-1.05)	0.064	0.45 (0.17-1.18)	0.11

Actual examinee performance was significantly better on questions answered correctly versus incorrectly by GPT-3.5 (84.7 ± 13.4% versus 72.7 ± 21.8%; *P* < 0.001) and GPT-4 (84.0 ± 15.9% versus 69.7 ± 19.8%; *P* < 0.001; Table 4).

**Table 4:** Examinee performance compared to ChatGPT on the ASSH SAE. Values are given as the mean percentage of correct answers plus or minus the standard deviation. ^*^Statistically significant values with *P *≤ 0.05. ASSH, American Society for Surgery of the Hand; SAE, Self-Assessment Examination; GPT, ChatGPT

	GPT-3.5			GPT-4		
Examinee performance	Correct	Incorrect	*P*-value	Correct	Incorrect	*P*-value
All examinees	84.7 ± 13.4	72.7 ± 21.8	<0.001*	84.0 ± 15.9	69.7 ± 19.8	<0.001*
2021	85.7 ± 11.9	66.9 ± 23.9	<0.001*	83.1 ± 16.8	69.1 ± 21.8	<0.001*
2022	83.6 ± 15.0	78.0 ± 18.4	0.08	84.8 ± 15.0	70.4 ± 17.1	<0.001*

## Discussion

As AI continues to integrate into medical practice, the applications of LLMs such as ChatGPT will expand. For ChatGPT to take hold as a viable resource for trainee, surgeon, and patient education, the accuracy of the answers it provides must be scrutinized [24]. This study showed GPT-4 outperformed GPT-3.5 on the ASSH SAE by answering significantly more questions correctly overall (68.9% versus 58.0%), on the 2022 SAE (72.9% versus 55.9%), and more difficult questions (63.6% versus 48.8%). In a multivariable logistic regression analysis, GPT-4 was significantly more likely to answer questions correctly compared with GPT-3.5, while more difficult questions and non-Basic Science questions were associated with incorrect answers. Actual examinees outperformed both versions of ChatGPT, but GPT-4 substantially reduced the performance gap. While ChatGPT does not appear to be ready for immediate integration into medical education and patient care in its present form, the improved performance in the most recent version suggests it may not be far away. 

The observed improvement in performance from GPT-3.5 to GPT-4 in the present study is consistent with the findings for other orthopedic and plastic surgery examinations. Multiple authors have evaluated the performance of ChatGPT on the Orthopaedic In-Training Examination (OITE). Kung et al. [15] assessed the performance of GPT-3.5 and GPT-4 on the 2020-2022 OITE text-only questions. The authors showed GPT-4 consistently outperformed GPT-3.5 overall (73.6% correct answers versus 54.3%, respectively) and for each examination year. GPT-4 performed above the American Board of Orthopaedic Surgery (ABOS) Part I passing standard for each year, but GPT-3.5 did not meet the passing standard. While OITE scores correlate with passing the ABOS Part I Examination, direct comparisons of correct answers on each examination are not valid [25]. Additionally, the authors showed that GPT-4 was able to provide a verifiable source for 87.8% of questions with a median journal impact factor of 5.2, while GPT-3.5 only provided a source for 47.2% of questions [15]. Rizzo et al. [26] performed a similar study on the same OITE questions but included questions with images and figures despite the inability of ChatGPT to analyze such inputs. The authors demonstrated lower performance than the former study, but GPT-4 still outperformed GPT-3.5 (58.7%-67.6% correct versus 46.5%-50.2%, respectively). Like our study, the authors showed GPT-4 outperformed GPT-3.5 on more difficult questions, but no statistical comparisons were performed. GPT-4 performed at the level of a post-graduate year (PGY) 2 or 3 orthopedic resident, while GPT-3.5 performed at the level of a PGY-1. Finally, Massey et al. [16] compared the performance of GPT-3.5 and GPT-4 to orthopedic residents on the American Academy of Orthopaedic Surgeons ResStudy orthopedic examination question bank. The authors showed poor performance of ChatGPT on questions with images (GPT-3.5, 22% correct; GPT-4, 36% correct) and significantly better performance on text-only questions (GPT-3.5, 38% correct; GPT-4, 61% correct). Orthopedic residents performed better than either ChatGPT version on questions with (76% correct) and without (73% correct) images. The sum of the current literature and the findings in the present study indicate that ChatGPT has made significant improvements in performance across multiple versions, but the current untrained models do not provide enough accuracy to warrant their use for medical education or patient care.

**Figure 1:**
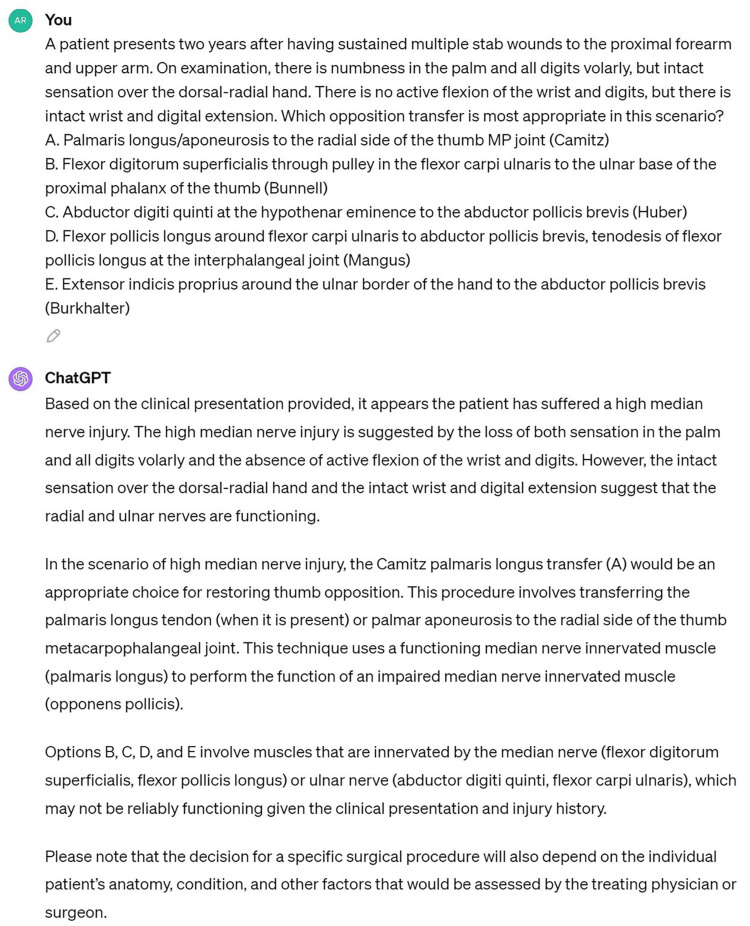
Example question from the 2022 ASSH SAE that was answered correctly by 94% of actual examinees, but GPT-4 incorrectly answered the question with incorrect logic. The question stem describes a high median and ulnar nerve injury. ChatGPT correctly identifies the median nerve injury, lists the muscles innervated by the median nerve, and then selects a muscle innervated by the median nerve as the tendon transfer option rather than the choice innervated by the intact radial nerve, the extensor indicis proprius. ASSH, American Society for Surgery of the Hand; SAE, Self-Assessment Examination

The accessibility of ChatGPT to the general population has led to investigations of open-ended questions to simulate common patient questions in hand surgery [11,12]. Crook et al. [11] evaluated the responses of GPT-3 to common patient questions regarding four common hand diagnoses, including carpal tunnel syndrome, cubital tunnel syndrome, trigger finger, and distal radius fractures. The authors showed the ChatGPT responses provided high-quality answers, but the utility of responses for patient use was limited by an advanced reading level and a failure to demonstrate uncertainty and nuance in some answers. ChatGPT also failed to provide references or source materials to determine where the information was obtained. Seth et al. [12] tested ChatGPT’s knowledge of carpal tunnel syndrome diagnosis and management with six questions and a simulated doctor-patient visit. While ChatGPT provided a logical, yet superficial, overview of the diagnosis and treatment options, it did not distinguish between nuances and frequently provided incorrect or nonexistent references when prompted for evidence. The authors acknowledged the potential use of AI for superficial patient inquiries but emphasized that integration of ChatGPT into patient care will require training on a hand surgery dataset, an improved ability to provide real and accurate references, and rigorous validation by experts. In the present study, ChatGPT was forced to select the single best answer, which frequently led to incorrect logic (Figure 1). Like Crook et al. [11] and Seth et al. [12], we showed ChatGPT is better at answering direct recall and basic science questions, while questions requiring clinical reasoning were more likely to be answered incorrectly. Han et al. [19] revealed that the GPT-3.5 model correctly answered 39.2% of the questions from the 2004 to 2013 ASSH SAE exams. In contrast, Ghanem et al. [18] focused exclusively on the GPT-4 model and its performance relative to a subset of 322 physicians on the 2019 ASSH SAE, noting a 65.83% correct response rate on text-based questions. The lower performance observed for earlier examination years may indicate that ChatGPT can use recent hand surgery data more readily than data that is 10 or more years old. Our findings similarly showed an improved performance of GPT-4 on the 2022 ASSH SAE compared to the 2021 exam. Furthermore, neither of the previous studies conducted a comparative analysis between the different versions of ChatGPT (GPT-3.5 versus GPT-4) or between ChatGPT and the full cohort of human participants for any given year. Our research fills this gap, providing insight into the incremental improvements being achieved in AI technology.

Both ChatGPT versions used in the present study achieved scores above the 50% cutoff to receive CME and MOC credit for orthopedic and plastic surgeons, but not the 75% threshold for general surgeons [17]. It should be noted that the passing thresholds for the ASSH SAE have been arbitrarily selected by the ABOS, the American Board of Plastic Surgery, and the American Board of Surgery [17]. Attaining a passing score does not necessarily correlate with performance on the Subspecialty Certificate in Surgery of the Hand. The significantly better performance of actual examinees on questions answered correctly versus incorrectly by ChatGPT shows humans and AI have similar difficulties while answering questions. The exclusion of questions with associated clinical images, radiographs, tables, and other media may inflate the performance of ChatGPT on the ASSH SAE, as shown by prior studies that included such questions [16,26]. With newer versions of ChatGPT, future studies should determine if media can be correctly interpreted in the context of clinical scenarios. Such functionality is essential for ChatGPT to become integrated into patient care and the education of hand surgeons.

This study had multiple limitations. First, ChatGPT's answers were limited to the information on which it was trained through July 21, 2023. Updates to the AI model beyond this date could impact the performance of ChatGPT but were not evaluated in this study. Second, we only included text-based questions given GPT-3.5 cannot analyze images. While newer versions of GPT-4 do allow for image inputs, this would not allow for comparisons to GPT-3.5. A recent study evaluating the performance of GPT-4 on the OITE recently showed poor performance on image-based questions with the new image processing feature of ChatGPT [27]. This suggests image processing is not ready for mainstream use, especially given the importance of interpreting radiographs, advanced imaging, and clinical pictures to make decisions in hand surgery. The lack of such inputs for ChatGPT is a major limitation to meaningfully integrating the technology into patient care. Third, the present study only evaluated a binary outcome of ChatGPT’s output - correct or incorrect - rather than assessing the logic behind its response. Fourth, we acknowledge that statistical significance is not synonymous with clinical significance. In our study, we observed an 11% improvement in performance from GPT-3.5 to GPT-4. The standard deviation for actual examinees was 18.3%. While there is no minimal clinically important difference (MCID) for the ASSH SAE, the 11% difference observed between ChatGPT versions exceeds a half standard deviation (9.2%) of the performance by actual examinees. The half standard deviation difference is an accepted threshold when no MCID exists in patient outcomes research [28]. This finding suggests that GPT-4 outperforms GPT-3.5 from a statistically and clinically important difference perspective. Additionally, the half standard deviation threshold would suggest both versions of ChatGPT perform worse than actual examinees by a clinically significant margin; however, direct statistical comparisons cannot be made between ChatGPT and actual examinees (GPT-3.5, -21.6%; GPT-4, -10.7%). Given the passing standard to receive CME and MOC credit from the ABOS and ABPS is 50%, however, both versions of ChatGPT exceeded this standard. Finally, future studies should determine if ChatGPT presents explanations that mirror the learning objectives of the ASSH SAE and whether the references provided are accurate and relevant. The tendency of ChatGPT and other LLMs to generate plausible, yet inaccurate, responses has been termed the hallucination effect [29]. In a domain where evidence can be nuanced, the expertise of a seasoned hand surgeon is essential to identify the most appropriate answers to complex questions.

## Conclusions

In conclusion, this study shows GPT-4 outperformed GPT-3.5 on the ASSH SAE, especially for more difficult questions. As AI and ChatGPT become more prevalent in medicine, hand surgeons should be familiar with the technology’s strengths and weaknesses. If ChatGPT is to play a major role in the education of trainees, surgeons, and patients, the models must be trained appropriately, and outputs scrutinized for accuracy, reliability, and consistency. Future studies should focus on training *chatbots* to improve the responses to common and complex hand surgery questions. In its current form, ChatGPT is not ready for unsupervised use in the clinical setting.
